# Peripheral giant cell granuloma as a sign of hyperparathyroidism in a patient under hemodialysis: A case report and review of literature

**DOI:** 10.1002/ccr3.7823

**Published:** 2023-08-21

**Authors:** Marzieh Yousefian, Arezoo Aghakouchakzadeh, Sajad Torki

**Affiliations:** ^1^ Department of Oral and Maxillofacial Medicine, School of Dentistry Alborz University of Medical Sciences Karaj Iran; ^2^ Department of Oral and Maxillofacial Pathology, School of Dentistry Alborz University of Medical Sciences Karaj Iran; ^3^ School of Dentistry Alborz University of Medical Sciences Karaj Iran

**Keywords:** giant cell granuloma, hemodialysis, hyperparathyroidism, parathyroid hormone, peripheral giant cell granuloma

## Abstract

Peripheral giant cell granuloma (PGCG) is a benign reactive exophytic oral lesion that originates from the periosteum or the periodontal ligament. It exclusively develops on the gingiva or alveolar mucosa. Hyperparathyroidism (HPT) is a possible etiology for its development. HPT is an endocrine disorder characterized by increased secretion of the parathyroid hormone (PTH). This case report describes a case of recurring PGCG in a patient diagnosed with secondary HPT after paraclinical assessment.

## Graphical abstract

PGCG as a sign of hyperparathyroidism.

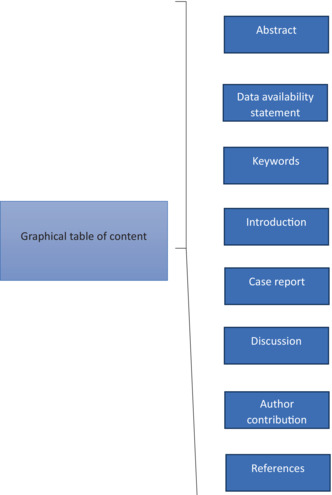

## 1 INTRODUCTION

Peripheral giant cell granuloma (PGCG) is a common, benign, reactive, non‐odontogenic exophytic oral lesion,^1^ which was first described by Jaffe in 1953.^2^ Local stimulatory factors such as food impaction, calculus, surgery, and periodontal disease are correlated with development of PGCG.^3^ Some other suggested etiologies for PGCG include xerostomia, poor oral hygiene, history of tooth extraction, and hormonal imbalance as in hyperparathyroidism (HPT).^4^ PGCG may originate from the periodontal ligament or the alveolar bone periosteum in both dentate and edentulous areas.^5^ In case of development of PGCG around a tooth, it probably originates from the periodontal ligament^1^; while, lesions developed at edentulous areas or around dental implants definitely originate from the periosteum and cortical bone.^6^

The clinical manifestations of PGCG are similar to those of other proliferating gingival lesions. Thus, its definite diagnosis should be made through histopathological analyses.^7^ The list of differential diagnoses of PGCG includes peripheral odontogenic fibroma,^8^ peripheral ossifying fibroma, hemangioma, pyogenic granuloma, and metastatic carcinoma, which have similar clinical manifestations but are histologically different, and have different recurrence rates as well.^7^

HPT is an endocrine disorder characterized by increased secretion of the parathyroid hormone (PTH). It was first described by von Recklinghausen in 1891, and is divided into three categories of primary, secondary, and tertiary HPT depending on its main origin.^9^

Osteoclastic bone resorption is a well‐recognized late outcome of HPT, which is characterized by lytic lesions in bones as in the jawbone.^10^ Other radiographic manifestations of HPT may include loss of lamina dura around the roots and loss of normal trabecular pattern of bone.^11^

PGCG has been reported as a rare oral manifestation of HPT. Although PGCG is not a common primary manifestation of HPT, HPT should be considered as an etiological factor when there are multiple lesions or in cases with recurrence after treatment.^12^

This case report describes a rare case of PGCG in a patient with HPT. Informed consent was obtained from the patient. A comprehensive search of the relevant literature was also conducted on case series, case reports, and review articles published in English by searching the PubMed, Scopus, Science Direct, and Google Scholar databases from January 2000 to December 2022.

## 2 CASE REPORT

A 38‐year‐old Iranian male patient presented to the Oral Medicine Department of School of Dentistry complaining of an intraoral lesion in the right side of the mandible which developed 1 year earlier. The patient had a history of kidney transplantation due to end‐stage renal disease because of using a lot of proteins for sport 19 years earlier, and experienced transplant rejection 2 years ago. He has been undergoing hemodialysis since then. Before getting the second kidney transplantation, he came for dental checkup and we found an intraoral lesion. The patient reported enlargement of the lesion within the past year. Clinically, the lesion was in the form of a sessile exophytic mass with an erosive surface and was located distal to tooth #45. The lesion measured 1 × 1 cm in size and was pink in color. A periapical radiograph was obtained from the area, which revealed bone erosion at the alveolar ridge crest distal to tooth #45 under the lesion.

**FIGURE 1:**
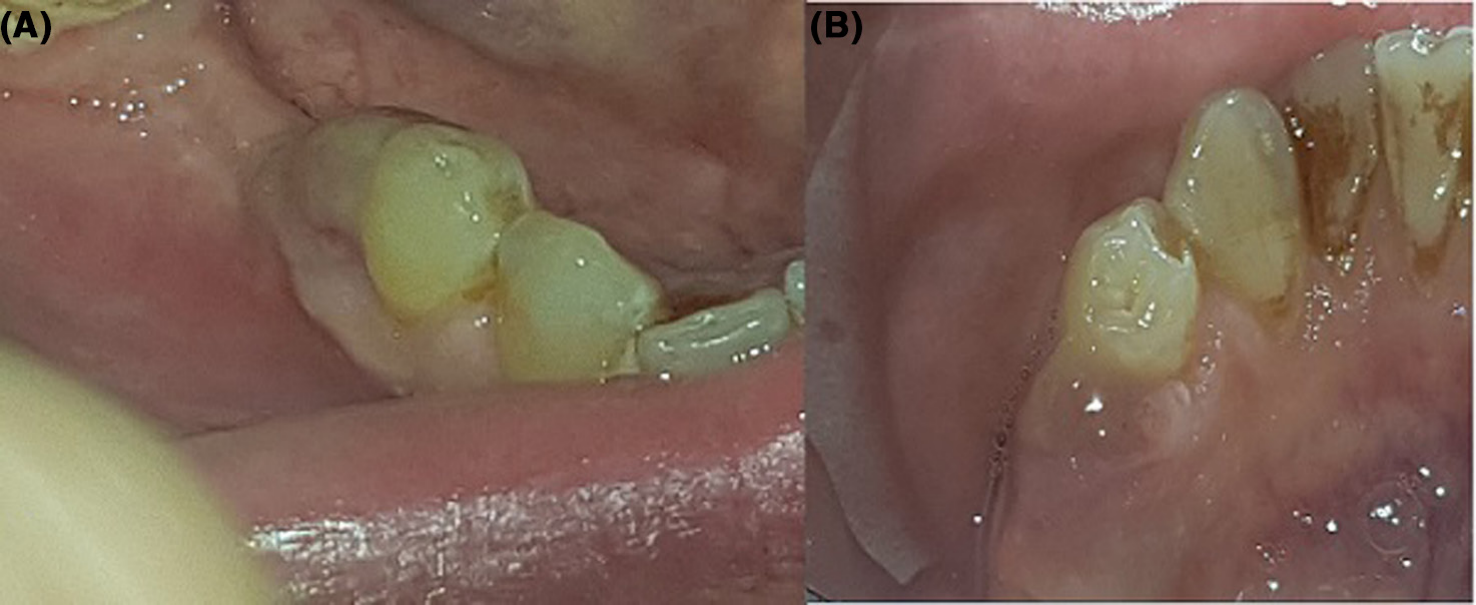
Clinical intraoral view of the lesion as a nodular exophytic mass with an erosive surface and pink color distal to tooth #44.

The patient underwent excisional biopsy and surgical extraction of tooth #45 with the differential diagnoses of pyogenic granuloma, peripheral ossifying fibroma, and PGCG. The excised lesion was sent for histopathological analysis, and the pathology report confirmed the diagnosis of PGCG. The lesion recurred distal to tooth #44 after 1 month with the same clinical appearance (Figure 1A,B).

**FIGURE 2:**
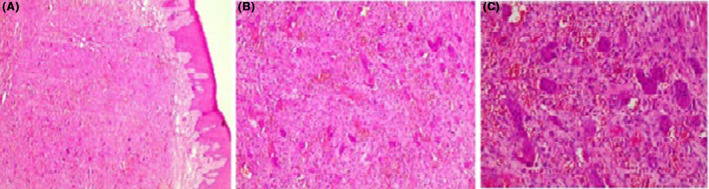
(A) Fibrovascular mass covered by parakeratotic stratified squamous epithelium (4x). (B) Multinucleated giant cells in the background of plump ovoid and spindle‐shaped mononuclear stromal cells (10x). (C) Numerous giant cells of various shapes and sizes and red blood cell extravasation (40x).

The lesion underwent excisional biopsy and the pathology report was as follows: “Microscopic examination showed a fibrovascular mass covered by parakeratotic stratified squamous epithelium (Figure 2A). The most characteristic feature was the proliferation of multinucleated giant cells within a background of ovoid and spindle‐shaped mesenchymal cells (Figure 2B). Also, hemosiderin pigmentation and extravasation of red blood cells were seen in some areas (Figure 2C).”

Considering the nature and behavior of the lesion, a parathyroid hormone (PTH) test was requested for the patient, and the result showed high level of PTH, indicating HPT. Thus, the patient was referred to an endocrinologist for further assessment and treatment. Considering the patient's conditions, his renal transplantation was postponed to after management of his HPT. At present, the patient's HPT is under control, he has undergone another renal transplantation, and the lesion has not recurred so far.

## 3 DISCUSSION

This study aimed to do a comprehensive review on case reports, case series, and review articles in English using the keywords “hyperparathyroidism,” “giant cell granuloma,” and “peripheral giant cell granuloma” on the occurrence of PGCG simultaneous with HPT and also reported a rare case of PGCG along with secondary HPT. The results are reported in Table 1.

**TABLE 1:** Demographic information of patients and characteristics of PGCG associated with HPT in the reviewed articles.

	Author	Age (years.)	Sex	Location	Medical/drug history
A	Naina Pattnaik et al	40	M	Anterior mandible (#32‐#33)	Primary hyperparathyroidism
B	Flavia Pirih et al	91	F	Posterior mandible (#35)	Lymphoma, bisphosphonate (actonel), and teriparatide
C	Christopher Choi et al	22	M	Left retromolar pad	End stage renal disease(ESRD), hypertension, hepatitis B carrier
D	Limongelli et al	Mean:57 ± 2	F:11 M:5	Maxilla:102 Mandible:138	N/R
E	Reid Lester et al	Mean: 45.76	F:125 M:115	Mostly in the posterior mandible	N/R

Abbreviations: M, male; F, female; N/R, not reported.

PGCG is an intraoral non‐neoplastic tumor‐like mass that develops specifically on the gingiva and edentulous alveolar mucosa, and can have various sizes.^13^ It can be pedunculated or sessile, and may vary in color from deep red to bluish red. It also bleeds easily.^14^ Its appearance may range from a smooth mass with well‐defined regular borders to a multi‐lobulated mass with an irregular shape and superficial depressions. The mandibular premolar–molar region is the most common site of development of PGCG.^15^ The rate of recurrence of PGCG varies from 5% to 70.6%^16^ with an incidence peak in the age range of 30–40 years.^13^

This lesion usually originates from the periosteal connective tissue or periodontal ligament^17^ probably in response to local stimuli.^18^ Also, hormonal stimulations^19^ particularly by estrogen are often involved in its occurrence, which may explain its higher prevalence in females.^5^ In rare cases, PGCG is the only manifestation of HPT in patients^20^ such that in a study by Boffano et al., of 874 patients with PGCG, only 28 had HPT, and PGCG was the only manifestation of their HPT.^21^

HPT refers to increased secretion of PTH which can be divided into four categories. Primary HPT refers to irregular overproduction of PTH due to a single adenoma in the parathyroid gland (85%) or disseminated hyperplasia of one or more parathyroid gland(s) (15%).^22^ The clinical manifestations of primary HPT may vary from an asymptomatic condition to a symptomatic classic disease with renal and/or skeletal complications.^23^ The prevalence of primary HPT in association with giant cell granuloma lesions is reportedly 5.9%.^9^

Overproduction of PTH in secondary HPT occurs due to chronic abnormal stimulation, such as low serum calcium level due to a renal disease or vitamin D deficiency. The majority of patients who undergo hemodialysis due to a renal problem have some degrees of HPT. The tertiary form of HPT is related to spontaneous and excessive release of PTH, which occurs after kidney transplantation in patients with chronic secondary HPT. The fourth type of HPT occurs due to ectopic production of PTH by malignant tumors.^22^

PGCG is also known as giant cell epulis, peripheral giant cell reparative granuloma, peripheral giant cell tumor, giant cell hyperplasia, and reparative giant cell granuloma.^24^ Approximately 7% of benign tumors of the jaw are PGCG.^25^ Although PGCG has the lowest prevalence among nodular gingival lesions such as pyogenic granuloma, peripheral ossifying fibroma, and fibrous dysplasia, it is a common giant cell tumor in the oral cavity.^17^

Histopathologically, fibroblasts are the baseline elements of these lesions. Multinucleated giant cells are abundantly found among the fibroblasts, along with some occasional islands of metastatic bone. Abundant blood vessels along the hemorrhagic area are also seen accompanied by the presence of hemosiderin and inflammatory cells in a cellular connective tissue background.^13^

The origin of multinucleated giant cells is not known. Some researchers believe that they show immunohistochemical properties of osteoclasts while some others suggest that they originate from the mononuclear phagocytic system. Some other possible sources include osteoblasts, epithelial cells, and spindle‐shaped cells.^26^

Search of the literature by the authors of the present study revealed association of PGCG and HPT in only a small number of cases, and their simultaneous occurrence is a rare phenomenon. Finally, it may be concluded that despite the rarity of association of PGCG and HPT, PGCG patients should be preferably assessed for HPT especially in cases with recurring lesions.

## AUTHOR CONTRIBUTIONS

**Marzieh Yousefian:** Supervision; writing – original draft. **Arezoo Aghakouchakzadeh:** Writing – original draft. **Sajad Torki:** Writing – original draft; writing – review and editing.

## FUNDING INFORMATION

This study has not any funder.

## CONFLICT OF INTEREST STATEMENT

Authors declare that there is no conflict of interest.

## CONSENT STATEMENT

A written informed consent was obtained from the patient.

## Data Availability

The data supporting this case report's findings are available from the corresponding author upon request.
